# The DNA binding parvulin Par17 is targeted to the mitochondrial matrix by a recently evolved prepeptide uniquely present in Hominidae

**DOI:** 10.1186/1741-7007-5-37

**Published:** 2007-09-17

**Authors:** Daniel Kessler, Panagiotis Papatheodorou, Tina Stratmann, Elke Andrea Dian, Cristina Hartmann-Fatu, Joachim Rassow, Peter Bayer, Jonathan Wolf Mueller

**Affiliations:** 1 Department of Structural and Medicinal Biochemistry, Center for Medical Biotechnology – ZMB, University of Duisburg-Essen, 45117 Essen, Germany; 2 Institut für Physiologische Chemie, Ruhr-Universität Bochum, 44780 Bochum, Germany

## Abstract

**Background:**

The parvulin-type peptidyl prolyl *cis/trans *isomerase Par14 is highly conserved in all metazoans. The recently identified parvulin Par17 contains an additional N-terminal domain whose occurrence and function was the focus of the present study.

**Results:**

Based on the observation that the human genome encodes Par17, but bovine and rodent genomes do not, Par17 exon sequences from 10 different primate species were cloned and sequenced. Par17 is encoded in the genomes of Hominidae species including humans, but is absent from other mammalian species. In contrast to Par14, endogenous Par17 was found in mitochondrial and membrane fractions of human cell lysates. Fluorescence of EGFP fusions of Par17, but not Par14, co-localized with mitochondrial staining. Par14 and Par17 associated with isolated human, rat and yeast mitochondria at low salt concentrations, but only the Par17 mitochondrial association was resistant to higher salt concentrations. Par17 was imported into mitochondria in a time and membrane potential-dependent manner, where it reached the mitochondrial matrix. Moreover, Par17 was shown to bind to double-stranded DNA under physiological salt conditions.

**Conclusion:**

Taken together, the DNA binding parvulin Par17 is targeted to the mitochondrial matrix by the most recently evolved mitochondrial prepeptide known to date, thus adding a novel protein constituent to the mitochondrial proteome of Hominidae.

## Background

Peptidyl prolyl *cis/trans *isomerases (PPIases, EC 5.2.1.8) play a role in protein folding and function by twisting the backbone of target proteins. PPIases are abundantly expressed in virtually all organisms and all cellular compartments [1-3]. One subclass of PPIases are parvulins, a group of enzymes involved in mitotic regulatory mechanisms and cell proliferation [4-8]. The human genome contains two parvulin genes. One encodes the well studied mitotic regulator Pin1, which isomerizes phosphorylated Ser/Thr-Pro motifs [9] and thereby is involved in a variety of cellular processes and conditions such as cell cycle regulation, cancer and Alzheimer's disease [10]; the other, the *Par14/PIN4 *locus on chromosome Xq13.1, gives rise to two protein species, Par14 and Par17 [8]. Par14 (also known as PIN4/EPVH/hPar14) [11,12] is assumed to be involved in cell cycle progression or chromatin remodeling [13-15]. Par14-like protein sequences are found in all multicellular organisms but are absent from yeasts [2]. Although Par14 cannot rescue the lethal phenotype of Ess1 deletion, which is the only parvulin-type PPIase in yeast [16], Uchida and co-workers proposed a compensating function for Par14 upon Pin1 inhibition or deletion in mammalian cells [7]. Par14 displays only very weak PPIase activity towards peptidic substrates [12] and hence might also perform cellular regulatory functions other than Pin1. The Par14 protein was initially detected within the nucleus, cytosol and mitochondria of human HEK 293 cells [11], but how this distribution was accomplished remained to be determined. Its distribution between the nucleus and cytosol [13,15,17] was described to be regulated in a phosphorylation-dependent manner [13] and deletion studies pointed to a nuclear import signal within the positively charged N-terminal section [15]. Within the nucleus, Par14 was reported to bind to pre-ribosomal ribonucleoprotein particles [17], and sequence-specifically to bent double-stranded DNA [15]. The basic N-terminal part was indispensable for high affinity DNA binding [15]. Such bent AT-rich segments of DNA are supposed to dictate nucleosome positioning [18] and play a role in transcription initiation.

Par17 is a recently described protein species [8] also encoded by the *Par14/PIN4 *locus on chromosome Xq13.1. Its elongated mRNA originates from alternative transcription initiation and occurs in all human tissues tested so far [8]. The 5'extension includes a 75 bp extended open reading frame whose expression was confirmed by *in vitro *translation reaction and by Western blotting of human HeLa and HepG2 cell lysates [8]. Due to its molecular mass of 16.6 kDa, the novel parvulin was denoted as Par17 following the *E. coli *Par10 and human Par14 nomenclature. The longer parvulin protein is encoded within the human genome but is absent from rodent, bovine and non-mammalian genomes [8].

The present study shows that Par17 occurs only in genomes from great apes species (Hominidae), but not in those of other primates, as shown by parvulin sequences from different primate genomic DNA samples. Par17 is directed to mitochondria by the novel N-terminal domain that functions as non-cleavable mitochondrial targeting peptide. The protein associates with mitochondrial surfaces and is transported into the matrix in a time and membrane potential-dependent manner. Par17 binds to double-stranded DNA at physiological salt concentrations as Par14 does. Par17 is thus a Hominid-specific DNA-binding constituent of the mitochondrial matrix that has not been detected in any recent mitochondrial proteomic studies. In addition, the prepeptide represents the most recently evolved functional mitochondrial targeting peptide known to date.

## Results

### Par17 occurrence in anthropoid genomic sequences

Within the human genome, the *parvulin/PIN4 *locus on chromosome Xq13.1 encodes an elongated open reading frame leading to the expression of Par17 [GenBank:NM_006223]. This elongation was already reported to be absent from bovine and rodent genomes [8]. The chimpanzee genome sequence [19] contained a gap at the corresponding locus preventing a comparison to other genomic parvulin sequences. Surprisingly, the macaque genome that became publicly available at that time did not contain an extended Par17. Therefore, further analysis of the occurrence of an elongated parvulin within the genomes of anthropoid species became of interest as Par17's extension must have been acquired during anthropoid evolution.

As the presequence was not encoded by an additional exon, but a 5' extension of exon 1, phylogenetic studies could be performed on genomic DNA eliminating the need for fresh primate material for mRNA isolation. Genomic DNA from 10 different anthropoid species covering New World monkeys (owl monkey and cotton-top tamarin), Old World monkeys (baboon, macaque and African green monkey) and apes (gibbon, orang-utan, gorilla, chimpanzee and human) were obtained from ECACC. These genomic DNA samples originated from stable anthropoid cell lines. Primers were designed to fit to primate sequences upon comparison of the human genomic sequence with the macaque genome. A fragment of the elongated exon 1 starting directly upstream of the Par17 ATG and including part of the Par14 coding sequence was PCR amplified from all these primate sequences. Exon 3 is conserved in all mammals including mouse, with only two amino acid exchanges between humans and rodents, and was used as positive control. Hence, genomic mouse DNA could serve as an ideal control for the existence of a parvulin gene (exon 3) and the absence of an elongation (exon 1). Uniform PCR products of the expected size were obtained in all reactions with genomic DNA templates, except for mouse exon 1; these products were all gel purified, cloned and sequenced. The human and macaque sequences for exon 1 as well as human, chimpanzee, macaque and mouse sequences for exon 3 were identical to the respective Ensembl genomic sequences.

**Figure 1:**
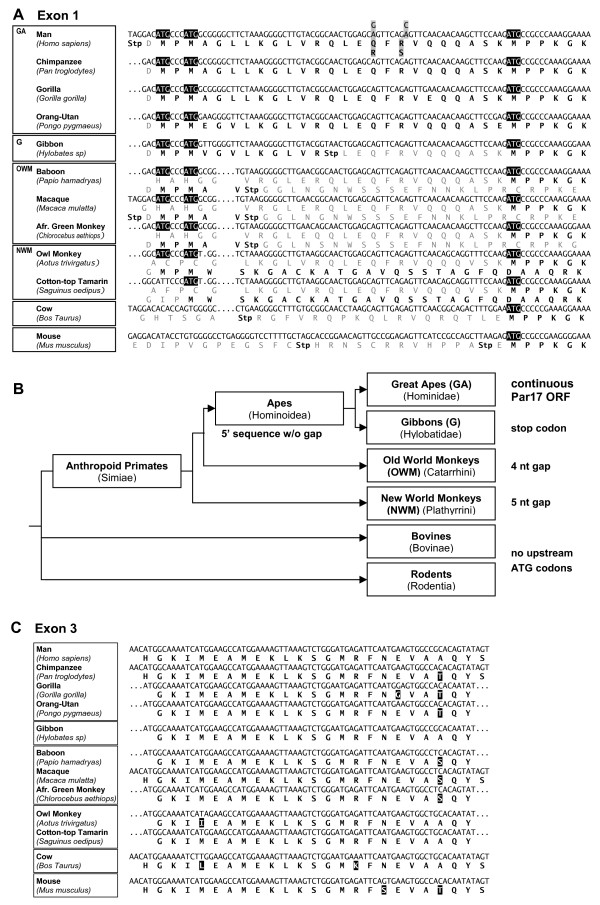
**Par17 is only encoded by genomic sequences of hominid species**. A. Sequence alignment of Par17 sequences. Parvulin exon 1 sequences were PCR amplified from genomic DNA of 10 different primate species. The resulting DNA sequences were aligned with respect to the Par17 and Par14 ATG codons; the respective *Bos taurus *sequence was obtained from Ensembl and aligned accordingly. Translated protein sequences in-frame with the respective ATG codons are given below the DNA sequences with stop codons written in red. Within the genomic sequences of *Homo sapiens *and *Macaca mulatta *(obtained from Ensembl), the Par17 ATG codons are immediately preceded by an in-frame stop codon. The Q16/R18 to R16/S18 coupled SNPs that were described for the human sequence are highlighted in grey; comparison with the other primate sequences reveals the QR form to be the ancestral one. Phylogenetic clades used in scheme B are indicated by boxes here and the respective abbreviations from B. B. Phylogenetic scheme of primate relationships and Par17 sequence features. The Par17 elongation is preformed in sequence in all anthropoid genomic DNAs tested. Additional ATG codons at the putative starting position of Par17 exist in all tested species, but are out-of-frame relative to the Par14 coding sequence in New World and Old World monkeys. The gap of five nucleotides in New World monkeys shrinks to a gap of four nucleotides in Old World monkeys, still preventing a continuous ORF with Par14. The upstream ATG codons are in-frame with the Par14 sequence in the clade of apes, only. Within the gibbon sequence however, an in-frame stop codon prevents expression of an extended protein. This stop codon has been sequenced in three independent clones. Thus, only Hominidae (*Homo, Pan, Gorilla *and *Pongo*) possess an extended ORF that can lead to the expression of Par17. (The length of the branches in this scheme does not reflect any phylogenetic distances.) C. Exon 3 sequences were obtained as in A. Exon 3 sequences are highly conserved with only two amino acid substitutions between human and cow or human and mouse, respectively. Amino acid exchanges relative to the human sequence are in white on black. Phylogenetic groupings are indicated by boxes as in A.

DNA sequence alignments together with putative translation products are shown in Figure 1A,C for the analyzed parts of exons 1 and 3, respectively, displaying high conservation within exon 3 and remarkable sequence changes within exon 1. The Par17 N-terminal domain is preformed in the DNA sequences of all anthropoids; additional ATG codons at the putative starting position of Par17 exist as well. However, they are out-of-frame in all Old World and New World monkey species. Several mutational rearrangements shift the presequence in-frame with Par14 allowing the expression of Par17 only in the Hominid sequences (*Homo, Pan, Gorilla *and *Pongo*). Although the two ATG codons are in-frame within the *Gibbon *sequence, an interspersed TAA stop codon prevents an elongated open reading frame. The sequence changes between different primate species are summarized in a schematic representation of their phylogenetic relationship (Figure 1B).

### The N-terminal extension of Par17 includes an amphipatic α-helix

**Figure 2:**
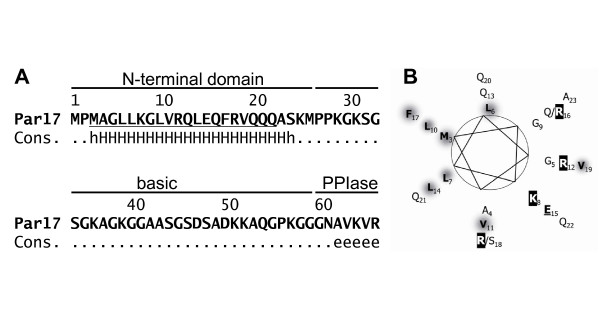
**The N-terminus of Par17 includes an amphipatic α-helix**. A. Secondary structure of Par17. The N-terminal Par17 sequence from Met3 to Ala23 is predicted to adopt α-helical conformation ("H") by different algorithms within the NPS prediction package, whereas the remaining part of this sequence is predicted to be random coil ("."). The PPIase domain starts at Asn61 with an extented beta-sheet ("e"). Consensus sequence prediction is based on HNNC, MLRC, PHD and Predator predictions. B. The N-terminal α-helix is amphipatic. Par17 protein sequence from Met3 to Ala23 is depicted as helical wheel with large, hydrophobic residues highlighted in grey, basic residues in blue and acidic ones in red.

The Par17 protein differs from Par14 within the N-terminus by 25 additional amino acids. The N-terminal Par17 sequence from Met3 to Ala23 is predicted to adopt an α-helical conformation (Figure 2A) by the secondary structure prediction package NPS [20]. When drawn as helical wheel, the amphipatic character of this part of Par17 becomes obvious, with Lys8, Arg12, Glu15, Arg18 and Lys25 on the one side and Met3, Leu6, Leu7, Leu10, Leu14 and Phe17 on the other (Figure 2B). The coupled SNPs (Arg16Gln [NCBI-SNP:rs6525589] and Ser18Arg [NCBI-SNP:rs7058353]) resulting in Q16R and R18S substitutions [8] within the predicted α-helix do not compromise the amphipatic character of the helix. We refer to these isoforms from now on as Par17-QR and -RS.

Positively charged amphipatic N-terminal α-helices are typical mitochondrial targeting signals [21]. Therefore, we predicted Par17's cellular localization using the online prediction tools Mitopred [22], Mitoprot [23], Predotar [24], PSortII [25] and TargetP [26]. The respective values for Par14, Par17-QR and -RS are given in Table 1. The 25 amino acid elongation is recognized as a mitochondrial targeting peptide by Mitoprot and Predotar, but not by PSORTII. By contrast, Mitopred classified Par14, Par17-QR and -RS as mitochondrial. As it was not possible to reliably predict Par17 localization by these prediction algorithms, it became necessary to investigate the intracellular localization of parvulin proteins experimentally.

**Table 1:** Predicted cellular localization of Par14 and Par17 proteins

**Program**	**Par14**	**Par17-QR**	**Par17-RS**
Mitopred [22]	0.846	0.99	0.99
Mitoprot [23]	0.0454	0.4088	0.9242
Predotar [24]	0.02	0.39	0.56
PSortII [25]	0.174	0.130	0.087
TargetP [26]	0.126	0.403	0.494

The output of sub-cellular localization prediction programs regarding the localization to mitochondria is given with numbers between 0 (no mitochondrial localization) and 1 (high probability of mitochondrial localization).

### Par17 is targeted to mitochondria within human cells

**Figure 3:**
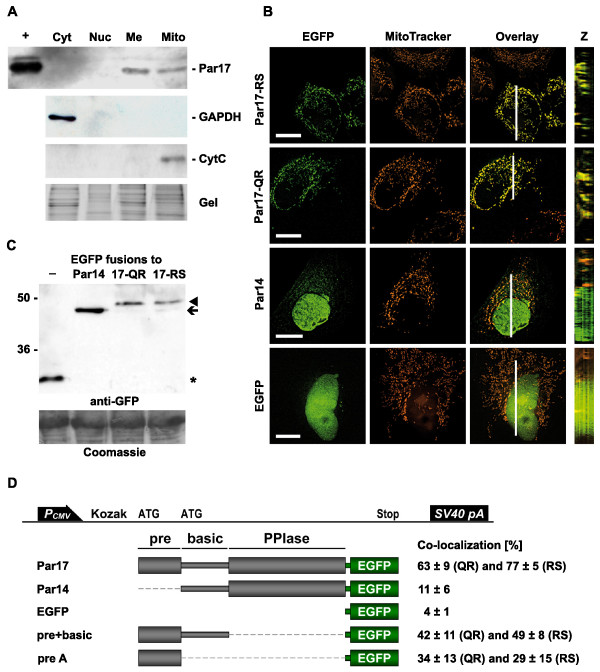
**Par17 is targeted to mitochondria in human cells**. A. Endogenous Par17 in sub-cellular fractions. HeLa cells were separated into cytoplasmic (Cyt), nucleic (Nuc), membrane (Me) and mitochondrial (Mito) fractions. Equal protein amounts were separated by SDS-PAGE, blotted onto nitrocellulose membranes and incubated with antibodies against the Par17 N-terminus or marker proteins. Par17 was detected as 17 kDa protein species within the membrane and mitochondrial fractions. Par17 without fusion tags expressed in *E. coli *BL21 from a pET-41 vector was used as positive control (+). B. Cellular localization of parvulin EGFP fusions. Par14, Par17-QR and -RS C-terminally fused to EGFP as well as EGFP without fusions were transfected into HeLa cells grown on cover slips, stained with MitoTracker and analyzed by fluorescence microscopy and 3D reconstitution. EGFP and MitoTracker fluorescence are shown as well as an overlay of these channels. White bar corresponds to 10 μm. The position of the Z stack image (3–4 μm in height) is indicated within the respective overlay image. A pronounced overlap between EGFP and MitoTracker signals is visible with the Par17-EGFP constructs that is not observed with Par14-EGFP or EGFP alone. C. Expression of the transfected constructs within HeLa cells as full length proteins. Anti-GFP Western blot and part of the Coomassie stained gel showing equal loading. All constructs were detected as full length protein species: Par17-EGFP (44.1 kDa, arrow head), Par14-EGFP (44.1 kDa, arrow) and EGFP (26.9 kDa, asterisk). D. Co-localization values of different EGFP fusion constructs. The fraction of EGFP fluorescence overlapping with the MitoTracker signal (in percent) was determined using the Cell^P co-localization tool for the fusion constructs shown in B. In addition, co-localization values for EGFP fusions of prepeptide and prepeptide coupled to the basic domain are displayed. All constructs containing the prepeptide were both tested as QR and RS variants. Co-localization values are expressed in percent ± standard deviation for more than 10 cells.

To determine the localization of the endogenous Par17 protein, an antibody against the N-terminal extension [8] was used for Par17 detection in sub-cellular fractions. HeLa cells were separated into cytosolic, nuclear, membrane and mitochondrial fractions, followed by Western blotting. Par17 was detected as a 17 kDa protein species within the mitochondrial and membrane fractions (Figure 3A). Purity of the mitochondrial fraction was demonstrated by blotting against cytochrome *c*. These data point to an association of endogenous Par17 with mitochondria or mitochondrial membranes.

To discriminate between Par17 and Par14 localization, overexpression studies using EGFP fusions were performed. EGFP was C-terminally fused to Par17-QR and -RS proteins and to Par14 as negative control. HeLa cells grown on cover slips were transfected with the above-mentioned constructs and stained with MitoTracker, a dye that accumulates in mitochondria with intact membrane potential. Fluorescence microscopic images of these constructs in HeLa cells are shown in Figure 3B; expression as full-length proteins was verified by Western blot using an anti-GFP antibody (Figure 3C). Par17-RS- and -QR-EGFP co-localized with mitochondrial staining. Totals of 77 ± 5 and 63 ± 9% of EGFP fluorescence were measured to overlap with the MitoTracker signal for Par17-RS and -QR, respectively. Par14-EGFP fluorescence is equally distributed throughout the cytosol and partially enriched in the nucleus, as has been described previously [15,17]. Co-localization values were far lower for Par14-EGFP (11 ± 6%) and EGFP alone (4 ± 1%).

Therefore, the presequence seemed to be the determinant for mitochondrial targeting, and the question arose whether the Par17 N-terminus was necessary and/or sufficient for mitochondrial targeting. To investigate this, EGFP was fused either to the presequence or to presequence coupled to the basic domain of Par17. Then, HeLa cells were transfected with these constructs. Their EGFP fluorescence overlapped with the MitoTracker signal to a lesser extent than the full length Par17 constructs with co-localization ratios of 49 ± 8% for presequence-RS-basic domain, 42 ± 11% for presequence-QR-basic domain, 29 ± 15% for presequence-RS and 34 ± 13% for presequence-QR. As can be deduced from these data, the mitochondrial association of Par17 is dependent on the presequence, but the basic and PPIase domains enhance mitochondrial targeting. The transfected constructs and co-localization values are schematically depicted in Figure 3D. Taken together, the cell fractionation studies and the localization of parvulin EGFP fusions provide independent evidence that Par17 isoforms are targeted to mitochondria.

### Par17 import into isolated mitochondria

**Figure 4:**
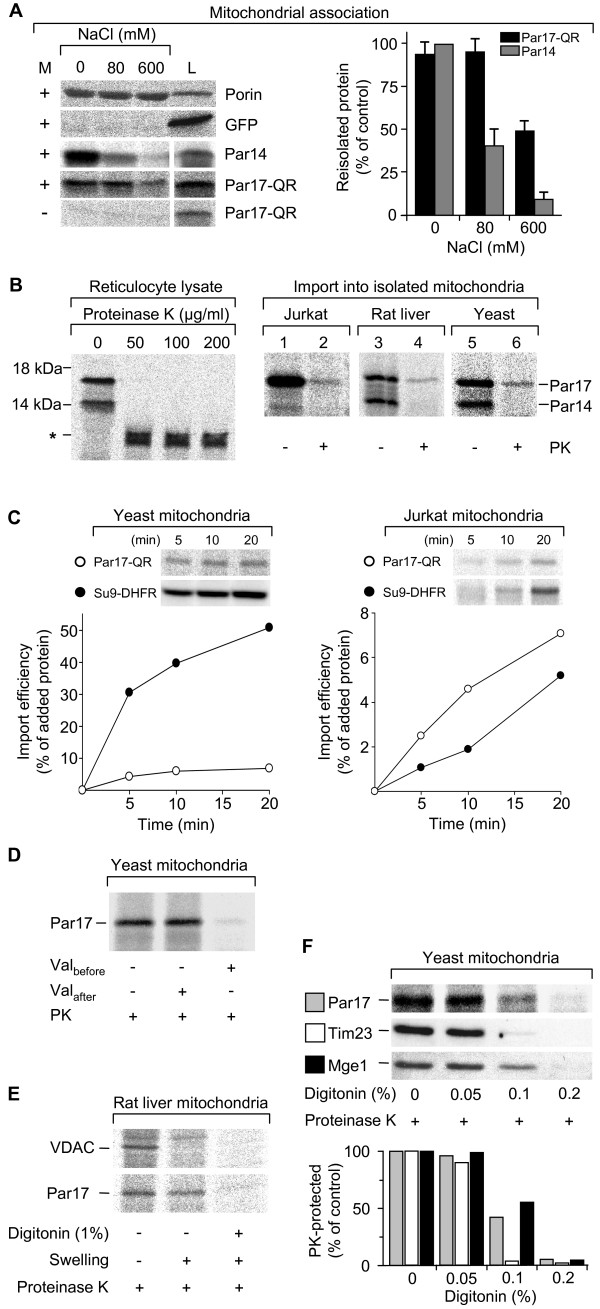
**Par17 import into isolated mitochondria**. A. Par17 and Par14 associate with mitochondrial surfaces in a salt-dependent manner. Yeast mitochondria were incubated with ^35^S-methionine labeled Par17 and Par14 lysates for 15 min at 25°C; Porin and GFP lysates were used as controls. Mitochondria were separated from soluble protein by the addition 500 mM sucrose buffer, subsequent centrifugation and additional washing in a 250 mM sucrose containing SEM buffer to ensure efficient separation of mitochondria from lysate ingredients. Mitochondria were then suspended in buffer containing increasing salt concentrations (0, 80 and 600 mM NaCl) and re-isolated by centrifugation. The samples within each panel including the respective reference sample (L, 20% for Porin and GFP, 50% for Par14 and Par17) were separated on the same gel to allow direct comparison (M +, mitochondria added). The amount of associated radioactively labeled protein was analyzed by SDS-PAGE and autoradiography. Mitochondrial association of Par14 and Par17 as displayed as bars on the diagram represent data normalized to the highest value including error bars (standard deviation for n = 3). B. Par17, but not Par14, is imported into isolated human, rat and yeast mitochondria. Left. Par17-QR and Par14 ^35^S-methionine labeled lysates were incubated with different amounts of PK showing complete degradation of soluble parvulin proteins at a PK concentration of 50 μg/ml; at least 100 μg/ml PK were used for all following experiments. Right. Radiolabeled Par17-QR was incubated with mitochondria from human Jurkat cells, rat liver or from yeast either with or without subsequent proteinase K (PK) treatment (at least 100 μg/ml for 10 min). Mitochondria were then re-isolated by centrifugation and analyzed by SDS-PAGE and autoradiography. Par17 reached a PK protected compartment irrespective of the source of mitochondria indicating mitochondrial import. Par14, also present in this reaction, could be re-isolated together with mitochondria during centrifugation, but did not reach a protease-protected compartment. C. Time dependence of Par17 mitochondrial import. Mitochondria from yeast and human Jurkat cells were incubated with Par17-QR lysates for the times indicated followed by PK treatment. To assess relative import efficiencies, lysates of Su9-DHFR (amino acids 1–69 from subunit 9 of *Neurospora crassa *ATP synthetase fused to mouse DHFR [28]) were imported into the same mitochondrial preparations for equal time intervals at identical reaction conditions. Import reactions of Par17-QR and mature Su9-DHFR were analyzed by autoradiography and densitometric quantifications. Values were expressed as percent of added protein with correction for the different numbers of methionine residues in the precursor and processed proteins of Su9-DHFR. D. Par17 import depends on the mitochondrial membrane potential. Import reactions were performed as in B (right panel). Mitochondria were treated with the uncoupling reagent valinomycin to dissipate the mitochondrial membrane potential either before or after import reaction. Par17 was only imported into mitochondria with intact membrane potential (without valinomycin; or when the uncoupling agent was added after import). E. Par17 reaches the inner membrane of rat mitochondria. Porin and Par17-QR were imported into mitochondria isolated from rat liver. The outer mitochondrial membrane was removed by hypo-osmotic swelling as indicated by the disappearance of the porin signal. The Par17 band however only disappeared upon complete lysis of mitochondria by digitonin indicating that Par17 was imported at least to the inner mitochondrial membrane. F. Par17 is imported into the mitochondrial matrix. Import reactions with yeast mitochondria were performed with increasing amounts of digitonin leading to stepwise lysis. The amounts of Par17-QR, the inner membrane protein Tim23 and the matrix protein Mge1 were monitored in parallel samples using autoradiography for Par17 and Western blotting for Tim23 and Mge1. Tim23 is already degraded at a concentration of 0.1% digitonin, while Par17-QR and the matrix protein Mge1 resist PK. Degradation of these two proteins is only observed upon addition of 0.2% digitonin indicating that Par17 is transported to the mitochondrial matrix. Gels (upper part) and densitometric analysis (lower part) are shown.

If the Par17 presequence represents a classical mitochondrial targeting signal, Par17 would be expected to be imported into mitochondria. However, a recent study reported cell type-specific mitochondrial association, but not import, of the related protein Pin1 [27]. Therefore, it was interesting to differentiate between association and import by *invitro *experiments with isolated mitochondria. As Par17-QR- and -RS-EGFP fusions co-localized with mitochondrial staining to the same extent within the range of error, the following *in vitro *experiments were performed with the QR isoform only. Par17 and Par14 were labeled by ^35^S-methionine incorporation and incubated with isolated yeast mitochondria. Soluble protein was separated from mitochondria and the mitochondrial pellet was subsequently washed with increasing salt concentrations followed by autoradiography (Figure 4A, left panel). As positive control, the yeast porin efficiently integrated into the outer mitochondrial membrane and therefore exhibited a salt-independent association with mitochondria, whereas GFP (as a negative control) was not able to associate with mitochondria at any salt concentrations. Par17 associated with mitochondria and could only be released to 50% upon washing with 600 mM NaCl, indicating tight association and/or import (Figure 4A, right panel). Interestingly, Par14 also associated at low salt concentrations with mitochondria, but was efficiently released from mitochondria at physiological or higher salt concentrations (80 mM NaCl corresponded to physiological conditions within this experimental buffer system [28]).

Next, mitochondria isolated from human, rat and yeast cells were used for proteinase K (PK) import studies, where all protein associated with mitochondria is re-isolated by centrifugation. Any protein that is not protected by translocation into sub-mitochondrial compartments will subsequently be degraded by PK. Although both Par14 and Par17-QR were degraded at 50 μg/ml PK (Figure 4B, left panel), at least 100 μg/ml PK were used throughout the following import studies. Par17 was re-isolated after incubation together with all types of mitochondria used (Figure 4B, right panel), again pointing to an association of the protein with mitochondrial organelles. Moreover, PK treatment showed that this labeled protein reached a protease-protected compartment, indicating import. Par14 also associated with mitochondria, but was not imported as it was completely degraded by PK.

Kinetic import experiments were then performed with different incubation times. Figure 4C shows the Par17 import into yeast and human mitochondria increased with time. As absolute import efficiencies cannot be compared between mitochondria from different sources, we used the fusion protein Su9-DHFR as positive control. Import into yeast mitochondria was significant, but rather low when compared to Su9-DHFR. However, Par17-QR and Su9-DHFR showed similar import efficiencies upon incubation with human mitochondria from the same preparation.

To test for the dependence on the mitochondrial membrane potential, yeast mitochondria were incubated with valinomycin either before or after the import reaction to dissipate the mitochondrial membrane potential. If import depends on the membrane potential, mitochondria with the de-coupling agent added before import would not import Par17 to the matrix. If this is not the case, Par17 should be imported irrespective of when valinomycin was added. As seen in Figure 4D, Par17 is only imported into mitochondria with intact membrane potential (lane 2 vs 3: addition of valinomycin after and before import, respectively). Therefore, the import of Par17 is dependent on an intact potential across the inner mitochondrial membrane.

To define the sub-mitochondrial localization of Par17 more precisely, a final set of import experiments were performed with reference proteins from different mitochondrial compartments. Par17 and the yeast porin, a component of the outer mitochondrial membrane were imported into rat mitochondria (Figure 4E). In a parallel reaction mitochondria were subjected to hypo-osmotic swelling, resulting in rupture of the outer membrane and loss of the porin protein. Par17, however, remained protected from PK degradation – indicating that it is at the very least transported to the inner mitochondrial membrane. It can only be degraded by PK when the mitochondria are lysed completely by the addition of 1% digitonin. The inner membrane protein Tim23 and the matrix protein Mge1, as controls for inner mitochondrial compartments, together with Par17 were then monitored during stepwise lysis of mitochondria using digitonin. Identical mitochondrial lysis reactions were performed for all three proteins with increasing amounts of digitonin, analyzed by autoradiography (Par17) or Western blot (Tim23 and Mge1). At a concentration of 0.1% digitonin the inner membrane protein Tim23 was already partially degraded, while Par17 and the matrix protein Mge1 resist PK degradation (Figure 4F). Tim23 is known to display one PK sensitive domain to the inter-membrane space, [29] which served as an epitope for the antibody used here. All three proteins became susceptible to PK degradation when the mitochondria were lysed completely by 0.2% digitonin.

Therefore, combined data obtained from *in vitro *experiments with yeast, rat and human mitochondria demonstrate the import of Par17 into mitochondria to be dependent on the presence of the novel N-terminal domain. In addition, Par17 was imported in a time- and membrane potential-dependent manner to the mitochondrial matrix, but without concomitant processing of the protein.

### Par17 binds to double-stranded DNA at physiological salt concentrations

**Figure 5:**
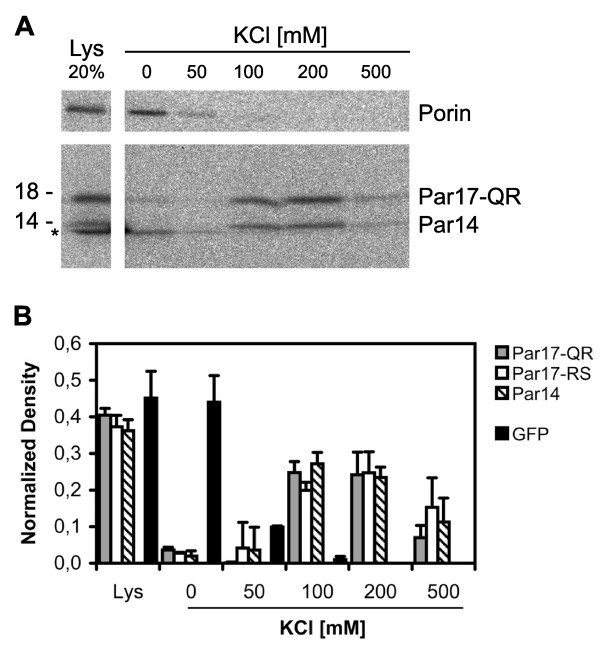
**Par17 binds to double-stranded DNA**. A. Binding of Par17 and Par14 to DNA. Radiolabeled Par17-QR and Par14 proteins were used for DNA cellulose binding assays. Following incubation of dsDNA with protein lysates, bound protein was eluted with increasing KCl concentrations. 20% of lysate (Lys) and KCl fractions were analyzed by SDS-PAGE and autoradiography. Asterisk (*), unspecific band caused by free label. Prominent signals for both, Par17 and Par14, are seen at 100 and 200 mM KCl. Porin was used as negative control and was washed off at 0 and 50 mM KCl. B. Quantitative analysis. Par17-QR, -RS and Par14 fused to EGFP as well as EGFP without fusions were subjected to the DNA binding assay. Blots were quantitatively analyzed by densitometry. Data from each experiment were normalized to their sum. Standard deviations are given from duplicate experiments. Par17 and Par14 proteins were eluted from dsDNA cellulose at 100, 200 and to a lesser extent at 500 mM KCl pointing to similar DNA-binding of the two parvulin proteins at physiological salt concentrations. EGFP without fusions was completely eluted from DNA cellulose at 50 mM KCl.

Par17 and Par14 exhibit different N-terminal regions and markedly different sub-cellular localizations. Therefore, the question arose whether Par17 can be involved in similar cellular functions as the nuclear-enriched Par14, yet within a different sub-cellular compartment: the mitochondrion. The highly conserved protein Par14 binds to AT-rich double-stranded DNA (dsDNA) within the nucleus [15], displays certain similarities to HMG proteins [13,15] and is assumed to be involved in transcriptional regulation. Hence, we tested whether binding characteristics to dsDNA were retained between the different parvulins. Binding of Par17-QR and Par14 to DNA cellulose was studied using radiolabeled lysates. Bound protein was eluted with increasing concentrations of KCl. Only at salt concentrations between 100 and 200 mM KCl was Par17-QR eluted from DNA cellulose (Figure 5A), with a residual Par17 fraction even eluting at 500 mM KCl. Par14 behaved in a similar manner. Porin was used as negative control for DNA cellulose binding and was already washed off with 0 and 50 mM KCl. A parallel DNA binding assay was performed with Par17-QR, -RS and Par14 as EGFP fusions expressed in HeLa cells. Gels were quantified by densitometry, with good agreement with the assay using radiolabeled proteins. From Figure 5B it can be derived that Par17-QR, -RS and Par14 all were comparably eluted from dsDNA at 100, 200 and even 500 mM KCl, whereas EGFP without fusion was completely eluted from the DNA cellulose beads at 50 mM KCl. Partial degradation of nucleic acids bound to the DNA cellulose beads used in these experiments by Benzonase diminished Par17 binding (data not shown). This shows that the Hominid-specific mitochondrial matrix protein Par17 can bind to double-stranded DNA at physiological salt concentrations.

## Discussion

### Import of Par17 into mitochondria

The recently described protein Par17 differs from the highly conserved parvulin Par14 only by a novel N-terminal domain. In this study, endogenous Par17 was detected in membrane and mitochondrial fractions, and EGFP fusions of Par17 were targeted to mitochondria. The dye used in these experiments (MitoTracker) is only accumulated by mitochondria with intact membrane potential. As no differing mitochondrial staining was detected for transfected cells as compared to untransfected ones, overexpression of Par17 seemed not to be toxic for HeLa cell mitochondria. EGFP fusions of the two Par17 isoforms, Par17-QR and -RS, were targeted to mitochondria with no significant differences in their co-localization values with MitoTracker. Therefore, only one isoform of Par17 (Par17-QR) was used for *in vitro *import experiments with isolated mitochondria; these experiments clearly showed human Par17 to be imported into the matrix of human, rat and yeast mitochondria. In addition, association of Par17 and Par14 proteins with mitochondrial surfaces was shown irrespective of the origin of the organelles. Consequently, most of the subsequent studies were performed with yeast mitochondria due to their experimental advantages [30], showing the import of Par17 to be dependent on intact mitochondrial membrane potential and to increase with time. Finally, sub-mitochondrial localization experiments showed that Par17 reached the mitochondrial matrix. Rulten et al [11] have already detected Par14 within the mitochondrial matrix using an antibody against the PPIase domain. However, as this domain is shared between the two proteins Par14 and Par17, the signal is likely to have originated from Par17 and not Par14.

One puzzling observation was that *in vitro *imported as well as endogenous Par17 within mitochondrial fractions was not processed after mitochondrial import, which would be expected for a classical targeting peptide. However, non-cleavable presequences have been described for the HSP60 chaperonin 10 [31], rhodanese [32], 3-oxoacyl-CoA thiolase [33] and the beta-subunit of the human electron-transfer flavoprotein [34]. Whether the negative charge of Glu15 within the amphipatic helix prevents this presequence being recognized by the matrix processing peptidase in analogy of yeast Leu9 [35] – another un-processed matrix protein with a glutamate at position 11 – remains to be tested. Even as a non-cleavable presequence, the N-terminal domain was shown to be necessary for import, but the basic and PPIase domains increase mitochondrial targeting.

### Mitochondrial proteomic studies

As a mitochondrial protein, Par17 should have previously been detected in one of the recently performed proteomic studies on the constituents of the mitochondrial proteome. Although the yeast mitochondrial proteome has been explored experimentally to about 80% coverage using a variety of mass spectroscopy and tagging techniques [36,37], neither Par14 nor Par17 are encoded by the yeast genome. Recent proteomic analysis of the yeast mitochondrial proteome (PROMITO) identified two novel PPIases (CPR1, [GenBank:NP_010439] and FPR1, [GenBank:NP_014264]) [38] with no sequence similarities to the parvulins described in this study. Mitochondrial proteomes of mammalian species are assumed to be considerably more complex [39], and their proportionate coverage by proteomic methods is correspondingly low [40,41]. The fraction of mitochondrial proteomes resisting identification by large-scale approaches tends to contain proteins that are expressed in low concentrations or only under certain conditions [42] as well as hydrophobic, small and/or highly basic proteins [41] with isoelectric points (pI) above 8. With an mRNA level about 50- to 100-fold lower than that of Par14 [8] and a pI of 9.96, Par17 hence represents a mitochondrial, low abundance, basic protein having escaped detection within mitochondria, even in a recent proteomic study on the human mitochondrial DNA nucleoid [43]. Peptides contained in either Par14 or Par17 have not been listed in any of the studies mentioned above.

### Evolution of the Par17 prepeptide within anthropoid primates

The proteome of mitochondria has changed dramatically during eukaryotic evolution, with proteins continuously recruited to the mitochondria expanding the proteome of this organelle and providing it with new functions or adaptations [42]. Mitochondrial recruitment can occur by exon duplication and exon shuffling, thereby exchanging existing targeting signals between different nuclear genes [42] as has been described, for example, for the *rps11 *gene from *Oryza sativa *[44] or the plant cytochrome *c*_1 _precursor [45].

The contrary seems to be the case with Par17. The evolution of Par17's presequence follows the evolution of primates as can be derived from the DNA sequences obtained in this study. Whereas the coding sequence for Par14 is highly conserved within all primates, the presequence of Par17 evolved steadily from New World monkeys to great apes. There is no spontaneous occurrence of a functional presequence as a result of an exchange with other nuclear encoded genes as this sequence motif is not found anywhere else in the human or macaque genomes. Taken the estimated time scales for primate evolution from Glazko and Nei [46] and Goodman et al [47], the insertion leading to the 5' ATG codons to be in-frame with the Par14 coding sequence was acquired not until the separation of Old World monkeys and apes about 23 ± 2 million years ago. Gibbons are known to have separated from great apes about 16 million years ago. The introduction of an interrupting TAA stop codon between the Par17 ATG and the Par14 ATG must have occurred after this branching point, but a more exact time point cannot be concluded from these data. Thus, phylogenetic comparison of genomic sequence data from 10 different primate species confines the Par17 protein to Hominids, only. The presequence therefore represents the most recently evolved functional mitochondrial targeting peptide known to date as it was not acquired until the divergence of apes and Old World monkeys.

Another issue that can be addressed in light of the different genomic Par17 sequences is the occurrence of the different human Par17 isoforms QR and RS. From the alignment in Figure 1A it can be derived that the isoform with Gln16 and Arg18 is the ancestral one. Why humans have acquired these coding SNP and if they serve any function remains to be elucidated in the future.

Could the 5' UTR of parvulin mRNAs have had a function for Par14 expression before it became a coding sequence? The partially conserved upstream ATG codons and short upstream open reading frames might have been involved in translational (down-) regulation of Par14 expression as has been described for other proteins [48,49]. In addition, differing mRNA secondary structures of the parvulin 5' UTR for several primates were predicted using the MFold prediction server [50]. Within the non-Hominid primate mRNAs, the sequence downstream of the first AUG codon is involved in an extended mRNA stem loop. This putative secondary structure is disturbed by the insertion leading to the sequence found in apes. Therefore, we speculate that mRNA secondary structure and/or translational regulation of Par14 was the evolutionary driving force for this part of the parvulin mRNA before it gained a protein coding function in great apes.

The evolutionary transition from apes to great apes involved significant remodeling of protein constituents of mitochondria: cytochrome *c *oxidase subunits [51,52] as constituents of the electron transport chain [53] as well as ATP synthetase subunits [54] have undergone accelerated changes during primate evolution. Par17 within the mitochondrial matrix now represents a novel protein constituent added to the Hominid-specific mitochondrial proteome.

### Par17 as mitochondrial DNA-binding protein

From the three PPIase families known, cyclophilinD and FKBP-38 have already been shown to be recruited to mitochondria where they play a role at the permeability transition pore and in apoptosis, respectively [55,56]. However, none of these PPIases is known to bind to double-stranded DNA (dsDNA). The present study however shows that Par17 is able to bind to dsDNA at physiological salt concentrations in a similar way to Par14. Assuming that sequence specificity is not altered between Par17 and Par14, the 16569 base pairs comprising human mitochondrial genome [GenBank:AC_000021] was searched for the following motifs that have been identified as tightest binders by Surmacz et al [15]: dsDNA octamers TAAAAAAT, ATGAAAAT and ATAAAAAT. As octamers, each of these motifs should statistically occur once in 65.5 kB of random sequence. As seven such motifs can be found within the human mitochondrial RefSeq DNA sequence, putative Par17 binding sites seem to be enriched within the mitochondrial genome. A function for Par17 in the context of mitochondrial DNA also seems likely due to its similarities to HMG proteins [13,15] as mitochondrial HMG proteins have been described very recently [57].

## Conclusion

The DNA binding parvulin protein Par17 is targeted to the mitochondrial matrix by the most recently evolved mitochondrial prepeptide known to-date only occurring in great apes species and man. Brain enlargement in primates has been paralleled by a significant remodeling of the mitochondrial proteome. Hence, a novel protein constituent of the Hominidae specific mitochondrial protein repertoire might have implications on the molecular understanding of brain diseases and the evolution of man.

## Methods

### Phylogenetic analysis of genomic sequences

Genomic DNAs of the following species were obtained from the European Collection of Cell Cultures (ECACC, Salisbury, Wiltshire, UK): *Pan troglodytes, Gorilla gorilla, Pongo pygmaeus, Hylobates sp., Papio hamadryas, Macaca mulatta, Chlorocebus aethiops, Aotus trivirgatus *and *Saguinus oedipus *. Human genomic DNA as a positive control was extracted from HeLa cells according to [58]. Genomic DNA from C57 BL6/J mice was kindly provided by Dr Manuela Wuelling (University of Duisburg-Essen, Essen, Germany). All genomic DNAs were amplified with a REPLI-g whole genome amplification kit (Qiagen, Hilden, Germany) prior to PCR. PCRs were performed using the primers 5'-cggctttcaggcatttgtttag-3' and 5'-tttccccgcttttccagaaccact-3' for amplification of exon 1 and 5'-gtcagacacattctatgtgaaaaac-3' and 5'-cccttgcctggctttatcttcact-3' for exon 3, respectively. PCR products were QIAquick gel extracted (Qiagen, Hilden, Germany), TOPO-TA cloned (Invitrogen, Karlsruhe, Germany) and sequenced (GATC Biotech, Konstanz, Germany). Sequences were edited and analyzed using the BioEdit software [59]. The Ensembl genome browser entries from human [Ensembl:ENSG00000102309], macaque [Ensembl:ENSMMUG00000012956], mouse [Ensembl:OTTMUSG00000018308] and chimpanzee [Ensembl:ENSPTRG00000022022] (exon 3 only) were used as reference sequences [60].

Parvulin exon 1 and exon 3 sequences obtained in this study have been deposited at EMBL with the following accession numbers: *Homo sapiens*, [EMBL:AM420633]; *Pan troglodytes*, [EMBL:AM420634]; *Gorilla gorilla*, [EMBL:AM420635]; *Pongo pygmaeus*, [EMBL:AM420636]; *Hylobates sp*., [EMBL:AM420637]; *Papio hamadryas*, [EMBL:AM420638]; *Macaca mulatta*, [EMBL:AM420639]; *Chlorocebus aethiops*, [EMBL:AM420640]; *Aotus trivirgatus*, [EMBL:AM420641]; *Saguinus oedipus*, [EMBL:AM420642]. *Chlorocebus aethiops *is denoted as *Cercopithecus sp*. within the respective database entry.

### Cell fractionation and Western blotting

For cell fractionation, HeLa cells were grown to confluence in petri dishes in medium containing D-MEM, 1× MEM and 1% FCS (all Gibco/Invitrogen, Karlsruhe, Germany) at 37°C and 7.5% CO_2_, trypsinized and washed in D-MEM. Cell pellets were separated into cytoplasmic, nucleic, membrane and mitochondrial fractions using the Mitochondria Isolation Kit (Qiagen, Hilden, Germany) according to the manufacturer's instructions. The nucleic fraction was resuspended in 200 mM Tris-HCl buffer, pH 8.8. Equal amounts of protein (30 μg) were separated on 15% SDS-PAGE gels and blotted on nitrocellulose membranes (Invitrogen, Karlsruhe, Germany) at 100 mA, 35 min. Following 30 min blocking with TBS-T 150 + 2% (w/v) dry milk, membranes were incubated overnight at 4°C with one of the following primary antibodies in TBS-T 150 + 2% milk: rabbit antiserum against the N-terminal Par17 extension (Ab-EXT, [8], 1:200 dilution); mouse anti-cytochrome *c *(clone 7H8.2C12, Abcam, 1:200); rabbit HRP-conjugated anti-GAPDH (Abcam, 1:500). The blots were then incubated with the respective HRP-conjugated secondary antibodies and developed using ECL kits (GE Amersham Bioscience, Freiburg, Germany) and CL-XPosure X-ray film (Perbio Science, Bonn, Germany).

### Construction and expression of EGFP fusion proteins

Coding sequences of Par14, Par17-QR, Par17-RS, QR presequence, RS presequence, QR presequence + basic domain and RS presequence + basic domain were PCR amplified using the following primers: 5'-aaaaaagaattcgccaccatgccgcccaaaggaaaaagtggt-3' (Par14 forward); 5'-aaaaaaggatcctttcttccttcgaccataataatatg-3' (Par14 and Par17 reverse); 5'-aaaaaagaattcgccaccatgcccatggcggggcttctaaag-3' (Par17 full length and presequence forward); 5'-atatatggatccttggaagcttgttgttgaacgctg-3' (Par17 presequence reverse); 5'-atatatggattcttgggaccttgagccttcttgtca-3' (Par17 presequence plus basic domain reverse). PCR products were *Eco*RI/*Bam*HI cloned into the pEGFP-N1 vector (Clontech, Saint-Germain-en-Laye, France) and were verified by sequencing.

For expression of parvulin EGFP constructs HeLa cells were grown on cover slips in 12-well plates in HeLa cell medium (as described above) to about 70–80% confluence. Transfections were performed using FuGene (Roche, Mannheim, Germany) according to the manufacturer's instructions and cells were allowed to grow for 40 h. Then the medium was removed and cells were washed in PBS pH 7.4 (Gibco/Invitrogen, Karlsruhe, Germany). For mitochondrial staining cells were incubated in a 100 nM solution of MitoTracker orange (Molecular Probes/Invitrogen, Karlsruhe, Germany) for 30 min, afterwards washed in PBS and fixed in PBS containing 3% paraformaldehyde and 0.2% Triton X-100 for 10 min. Cover slips were washed and mounted on microscope slides.

### Image acquisition and data analysis

Image data were retrieved and analyzed with an Olympus BX61 Microscope equipped with bandpass filter cubes, an UPlan SApo 100× Oil objective, an F-View II camera and the Cell^P software (all from Olympus, Hamburg, Germany, and SIS Soft Imaging Systems, Muenster, Germany). Deconvolution of Z-stacks was performed using the nearest neighbor deconvolution algorithm with a haze removal factor of 99%. Processing of the pictures and calculation of co-localization values were all performed within the Cell^P software. In addition, cells were analyzed with a Leica DM IRB Confocal Microscope with TCS SP 2 Confocal Scanner (Leica, Wetzlar, Germany) to judge the quality of the deconvoluted Z-stacks described above.

### Isolation of mitochondria

Mitochondria from yeast were isolated from wild type strain PK82 (MAT α, his4-713, lys2, ura3-52, trp1, leu2-3; [61] grown in YPG medium (1% (w/v) yeast extract, 2% (w/v) bacto-peptone, pH 5.0, containing 3% (v/v) glycerol) following standard procedures [28,30]. Rat liver and Jurkat cells mitochondria were obtained essentially as published previously [62,63].

### Import of proteins into isolated mitochondria

Radiolabeled Par17-QR, -RS, Par14 and control proteins were synthesized in rabbit reticulocyte lysate (TNT T7 Coupled Reticulocyte Lysate System, Promega, Mannheim, Germany) in the presence of ^35^S-labeled methionine (ICN Biomedical Research Products, Eschwege, Germany). In sample sizes of 50–100 μl the radiolabeled proteins were imported into isolated mitochondria as described previously [28]. Under standard conditions, the import assay contained BSA buffer (3% (w/v) BSA, 80 mM KCl, 10 mM MOPS-KOH, pH7.2), 2–5 μl reticulocyte lysate, 2 mM NADH, 1 mM ATP, 20 mM potassium phosphate and yeast mitochondria (30 μg mitochondrial protein). As import into isolated mammalian mitochondria is generally less efficient compared to yeast mitochondria [28,64], mammalian mitochondria were added at a higher concentration (40 μg mammalian mitochondrial protein). The import reactions were carried out at 25°C and protein uptake stopped by incubation at 0°C. Non-imported proteins were either degraded by addition of proteinase K at a final concentration of at least 100 μg/ml and incubation for 10 min at 0°C or the samples were left untreated. The protease was inactivated by addition of 2 mM PMSF and additional incubation for 5 min at 0°C. Eventually, the mitochondrial membrane potential was dissipated by addition of valinomycin (Sigma, Munich, Germany) at a final concentration of 1 μM from a 100-fold concentrated stock solution in ethanol. Selective opening of the mitochondrial outer membrane was achieved by incubation of mitochondria in EM buffer (1 mM EDTA, 10 mM MOPS-KOH, pH 7.2) for 20 min at 0°C (swelling). Alternatively, digitonin (Calbiochem/Merck, Darmstadt, Germany) was added up to a final concentration of 0.1% (w/v). For association studies, mitochondria were separated from soluble protein by the addition of SEM buffer (500 mM sucrose, 1 mM EDTA, 10 mM MOPS, pH 7.2) and subsequent centrifugation. An additional washing step in a 250 mM sucrose containing SEM buffer was performed to ensure efficient separation of mitochondria from lysate ingredients.

### DNA binding assay

HeLa cells were transfected with Par14-, Par17-RS- and Par17-QR-EGFP fusion constructs as described above and lysed in 50 mM K-PO_4_, 1 mM EDTA, 0.1% Triton X, 1 mM Pefablock (Roth, Karlsruhe, Germany), pH 7.0. Cells were filled up to a total volume of 4 ml with dilution buffer (20 mM Tris-HCl, pH 7.5, 10% glycerol, 1 mM EDTA, 1 mM DTT, 0.1% Triton X, 37.5 mM (NH_4_)_2_SO_4_), disrupted by ultrasonic and centrifuged to remove debris (20 min, 4°C, 4000 × g). DNA cellulose (0.25 g calf thymus DNA cellulose (Sigma, Munich, Germany)) was suspended in 3 ml binding buffer (20 mM Tris, pH 7.5, 10% glycerol, 100 mM KCl, 1 mM EDTA, 10 mM NaF and 0.1% Triton X) and incubated rotating at 4°C overnight. A total of 850 μl of cellulose suspension was transferred to a 2 ml microcentrifuge tube, washed with fresh binding buffer and centrifuged (10 min, 4°C, 2000 × g) three times. The supernatant was then replaced by 850 μl of cell lysate. The suspension was incubated with rotating for 1 h at 4°C and then centrifuged (2 min, 10000 × g, 4°C). The supernatant was stored at 4°C. The cellulose was washed successively with binding buffer containing increasing concentrations of KCl. After each washing step the suspension was incubated and centrifuged as described above. All supernatants were methanol/chloroform precipitated, run on 12.5% SDS-PAGE gels and analyzed by Western blotting using anti-GFP antibodies as described above. Blots were analyzed quantitatively by densitometry using Scion Image software [65].

The experiments were repeated using radiolabeled proteins instead of EGFP fusion constructs. Here, 50 μl of cellulose suspension and 5 μl of cell lysate as well as 50 μl of the different binding buffers were sufficient. All incubation times were reduced to 20 min. Gels were analyzed autoradiographically as described above.

## Abbreviations

EGFP, enhanced green fluorescent protein; dsDNA, double-stranded DNA; PPIase, peptidyl prolyl cis/trans isomerase; SNP, single nucleotide polymorphism; PK, proteinase K; HMG, high mobility group (protein); Par17-QR and -RS, (human) parvulin 17 with either Gln16+Arg18 or Arg16+Ser18; DHFR, dihydro-folate reductase; UTR, untranslated region

## Competing interests

The author(s) declare that they have no competing interests.

## Authors' contributions

DK performed analysis of genomic sequences and cell fractionation studies as well as EGFP transfections with microscopic analysis, and assisted in the writing. PP performed mitochondrial import studies as well as DNA binding studies and the respective data analysis. TS was involved in DNA binding studies and performed cloning and Western blotting studies. EAD isolated Jurkat and rat liver mitochondria and performed mitochondrial association studies. CHF recorded confocal images and participated in Western blotting. JR participated in the analysis and discussion of the *in vitro *import data. PB coordinated the investigations and participated in the design of the manuscript. JWM conceived and coordinated the study, analyzed data and wrote the manuscript. All authors read and approved the final manuscript.
